# Supplementing High-Density SNP Microarrays for Additional Coverage of Disease-Related Genes: Addiction as a Paradigm

**DOI:** 10.1371/journal.pone.0005225

**Published:** 2009-04-21

**Authors:** Scott F. Saccone, Laura J. Bierut, Elissa J. Chesler, Peter W. Kalivas, Caryn Lerman, Nancy L. Saccone, George R. Uhl, Chuan-Yun Li, Vivek M. Philip, Howard J. Edenberg, Stephen T. Sherry, Michael Feolo, Robert K. Moyzis, Joni L. Rutter

**Affiliations:** 1 Department of Psychiatry, Washington University School of Medicine, St. Louis, Missouri, United States of America; 2 Systems Genetics Group, Biosciences Division, Oak Ridge National Laboratory, Oak Ridge, Tennessee, United States of America; 3 Department of Neurosciences, Medical University of South Carolina, Charleston, South Carolina, United States of America; 4 Department of Psychiatry, University of Pennsylvania, Philadelphia, Pennsylvania, United States of America; 5 Department of Genetics, Washington University School of Medicine, St. Louis, Missouri, United States of America; 6 Molecular Neurobiology Branch, National Institutes of Health (NIH) - Intramural Research Program (IRP) National Institute on Drug Abuse (NIDA), Bethesda, Maryland, United States of America; 7 Center for Bioinformatics, National Laboratory of Protein Engineering and Plant Genetic Engineering, College of Life Sciences, Peking University, Beijing, China; 8 Department of Biochemistry and Molecular Biology, Indiana University School of Medicine, Indianapolis, Indiana, United States of America; 9 National Center for Biotechnology Information, National Library of Medicine, National Institutes of Health, Bethesda, Maryland, United States of America; 10 Department of Biological Chemistry, University of California Irvine, Irvine, California, United States of America; 11 Institute of Genomics and Bioinformatics, University of California Irvine, Irvine, California, United States of America; 12 Division of Basic Neuroscience and Behavioral Research, National Institute on Drug Abuse (NIDA), National Institutes of Health (NIH), Department of Health and Human Services(DHHS), Bethesda, Maryland, United States of America; University of Cape Town, South Africa

## Abstract

Commercial SNP microarrays now provide comprehensive and affordable coverage of the human genome. However, some diseases have biologically relevant genomic regions that may require additional coverage. Addiction, for example, is thought to be influenced by complex interactions among many relevant genes and pathways. We have assembled a list of 486 biologically relevant genes nominated by a panel of experts on addiction. We then added 424 genes that showed evidence of association with addiction phenotypes through mouse QTL mappings and gene co-expression analysis. We demonstrate that there are a substantial number of SNPs in these genes that are not well represented by commercial SNP platforms. We address this problem by introducing a publicly available SNP database for addiction. The database is annotated using numeric prioritization scores indicating the extent of biological relevance. The scores incorporate a number of factors such as SNP/gene functional properties (including synonymy and promoter regions), data from mouse systems genetics and measures of human/mouse evolutionary conservation. We then used HapMap genotyping data to determine if a SNP is tagged by a commercial microarray through linkage disequilibrium. This combination of biological prioritization scores and LD tagging annotation will enable addiction researchers to supplement commercial SNP microarrays to ensure comprehensive coverage of biologically relevant regions.

## Introduction

Genome-wide association studies (GWAS) have pushed human genetics into a new era. Advances in technology and affordability are rapidly allowing GWAS to identify genetic variants that affect risk for human disease (http://genome.gov/26525384). GWAS have used microarrays that allow parallel assessment of hundreds of thousands of single nucleotide polymorphisms (SNPs), and now also copy number variant (CNVs). The technology of Affymetrix (http://affymetrix.com), Illumina (http://illumina.com) and Perlegen (http://perlegen.com) have been most often used for these studies. While these microarrays have been designed to efficiently explore genetic variation across the entire human genome, they each provide better coverage in some genomic regions than in others [1], [2].

Most of the SNPs assessed by these commercial microarrays were chosen in ways that are not based on hypotheses about the underlying biology of any particular disorder. However, to the extent that there is a body of knowledge concerning the biology of a disorder, not all genes may be as likely, *a priori*, to contain disease associated variants. Thus, if a commercial microarray is used for a GWAS, we might ask – how well does the microarray cover biologically relevant genes for which there is *a priori* reason to believe their products are involved in the disease of interest [3]? For example, because genes that encode nicotinic cholinergic receptors have a clear biochemical connection to nicotine dependence, as do alcohol dehydrogenases for alcoholism, we should be especially vigilant in testing the hypothesis that variants in these genes might influence addiction vulnerability.

We have assembled data concerning the biology of addiction in order to examine the genomic coverage of commercial SNP microarrays. We show that several of these arrays, including top of the line models such as the Illumina 1M and Affymetrix 6.0, fail to cover a significant amount of common genetic variation in addiction-related genes. We have also developed a SNP database that can be used to supplement these microarrays to achieve comprehensive coverage of these regions. This database is annotated with numeric prioritization scores [4] indicating the biological relevance of a SNP to addiction. This allows the prioritization of supplementary SNPs when resources are limited. We also include annotation indicating the extent to which a SNP is tagged through linkage disequilibrium (LD) with some SNP on a specific array with respect to a specific HapMap population: African, Chinese, European-American and Japanese. By combining the prioritization scores with LD tagging data, we can determine the most biologically relevant SNPs for comprehensive LD tagged coverage of genes that are biologically relevant to addiction.

## Results

Table 1 shows the number of supplementary SNPs needed to tag all common variants in various populations for our primary set of 910 genes that are biologically relevant to addiction. This set includes 486 genes that were derived mainly through an expert nomination process, and 424 additional genes that correspond to roughly the top 5% of genes identified using mouse systems genetics (Chesler and colleagues, submitted). Together this set of 910 genes was our primary set for the analysis of microarray coverage (see supporting file S1 for the complete list of these genes). We assessed the SNP coverage of these genes by determining if common SNPs (MAF≥5%) were tagged through LD by SNPs on a particular microarray. In Table 1, for each array and each population, we report the number of common SNPs in these genes that fail to satisfy *r* ^2^≥0.8 with a SNP from the array; that is, the number of SNPs not tagged by the array. For example, we found that 57% of the common SNPs in these genes were not tagged by the Affymetrix 5.0 SNP microarray in the African population. In other words, due to the haplotype/LD structure in the African population, 43% of the common genetic variation in these regions fails to be captured by this microarray. Table S1 gives a broader view of how microarray coverage depends on biology, and shows that the Illumina coverage tends to improve with the prioritization score, while the Affymetrix coverage is uniform.

**Table 1:** The number of SNPs required to supplement commercial microarrays in order to comprehensively cover our primary set of 910 genes that are biologically relevant to addiction.

	Number of Supplementary SNPs (%)			
	African	Chinese	European-American	Japanese
*All Common SNPs:*	86,925	73,241	79,274	72,843
Microarray				
Affymetrix 5.0	49,762 (57)	24,691 (34)	28,001 (35)	24,183 (33)
Affymetrix 6.0	27,945 (32)	11,499 (16)	12,542 (16)	11,132 (15)
Illumina 300 Duo	56,475 (65)	22,821 (31)	16,364 (21)	22,934 (31)
Illumina 550	37,776 (43)	10,166 (14)	7,362 (9)	9,962 (14)
Illumina 610 Quad	36,448 (42)	10,064 (14)	7,324 (9)	9,845 (14)
Illumina 650Y	29,417 (34)	9,396 (13)	7,062 (9)	9,105 (12)
Illumina 1M	23,441 (27)	6,370 (9)	5,117 (6)	6,056 (8)

Results are listed for four populations. The numbers in parentheses are the percentages of all common SNPs in these genes in the corresponding population. For example, there are 86,925 SNPs in these genes with MAF≥5% in the African population, and we found that 57% of these SNPs fail to satisfy *r* ^2^≥0.8 with a SNP from the Affymetrix 5.0 microarray.

These results suggest that a significant amount of common genetic variation in these addiction related genes is unaccounted for by these commercial SNP microarrays. The deficiency is particularly high in the African sample. This is likely due to the lower LD in this older population, which means more SNPs are required for tagging. While the Illumina 1M clearly provides the best coverage, we would still need to add 23,441 SNPs to tag all common SNPs in the HapMap African sample for these 910 genes. The best-case scenario is when the Illumina 1M is used for European-Americans. But even in this case, there are still 5,117 SNPs that are not well represented.

Table 2 shows some examples of coverage by the Illumina 610 Quad microarray for ten genes that are of particular interest. We chose this array because it offers a median level of coverage among the seven arrays we studied. These genes were among the most highly prioritized by the addiction researchers with whom we consulted. The selection process involved a number of criteria, including pharmacogenetic pathways, gene expression data, and mouse models. For example, *CDH13* (Cadherin 13) is known to be expressed in neurons in the human adult cerebral cortex, midbrain, thalamus and medulla regions [5]. Because it is also known to inhibit neurite extension [6] and activate a number of signaling pathways [7]–[10], it is a strong candidate for the genetic study of addiction phenotypes [11]. *CDH13* contains 2,414 SNPs that are common in the African population, and only 50% of these are tagged by the by the Illumina 610 Quad microarray. Figure S1 shows the complete distribution of individual gene coverage percentages using our primary set of 910 genes for the Illumina 610 Quad microarray in each population.

**Table 2:** The number of SNPs required to supplement the Illumina 610 Quad microarray for genes of particularly strong interest.

Gene	Number of Supplementary SNPs (%)			
	African	Chinese	European-American	Japanese
*CDH13*	1,207 (50)	417 (21)	340 (15)	389 (20)
*CHRNA3*	7 (28)	2 (9)	0	0
*CHRNA5*	4 (15)	1 (5)	0	4 (21)
*CHRNB4*	4 (33)	5 (38)	3 (23)	5 (38)
*COMT*	11 (48)	4 (17)	4 (20)	3 (13)
*GABRA2*	32 (29)	5 (5)	8 (8)	5 (5)
*MAPK1*	20 (33)	7 (10)	0	16 (24)
*OPRM1*	90 (40)	20 (14)	16 (6)	13 (7)
*SLC1A2*	82 (36)	12 (5)	7 (3)	19 (9)
*SLC7A11*	19 (48)	7 (28)	7 (21)	3 (10)

The numbers in parentheses are the percentages of all common SNPs in these genes in the corresponding population.

We have designed a SNP database (available at http://zork.wustl.edu/nida/neurosnp.html) to systematically determine how to supplement these commercial microarrays for addiction. Our database includes a SNP prioritization score based on the genomic information network (GIN) method introduced by S. Saccone and colleagues [4]. This method was originally designed to systematically incorporate *a priori* biological hypotheses into the prioritization of SNPs after a genome-wide association study. The method begins with a set of SNPs that are ranked by their association *p*-values, and then increases the rank of a SNP when it is determined to be biologically relevant to the phenotype according to an *a priori* set of conditions, such as being in a biologically relevant gene, and additionally, perhaps, being a missense mutation. The score is a measure of biological relevance to addiction, and can be used independently of association *p*-values to prioritize which SNPs are selected to supplement commercial microarrays. The score incorporates SNP/gene functional properties (such as coding and promoter regions), human/mouse evolutionary conservation, and a quantitative trait locus (QTL) mapping method that utilizes mouse models to identify genes associated with addiction phenotypes (Chesler and colleagues, submitted). Figure S2 shows the distribution of prioritization scores for our genome-wide SNP database, and Figure S3 shows the GIN network model we used to model addiction, which was adapted from the nicotine dependence model used by Saccone and colleagues [4].

In addition to our primary set of 910 genes, the mouse systems genetics method (Chesler and colleagues, submitted) that identified 7,842 additional genes with potential biological relevance to addiction through mouse QTL and gene expression correlation analysis and the GIN prioritization scores reflect this quantitative assessment of biological relevance. Genes with a large number of mouse associations are prioritized more highly, and those with a relatively low number receive little increase in the prioritization score relative to arbitrary genes (see the methods section for details). These additional data provide a broader measure of biological relevance to addiction which may be useful for prioritizing SNPs for further study after a GWAS [4] or fine mapping a region of genetic linkage. This method has the effect of combining information from the expert nomination process and the mouse systems genetics data. SNPs in the 486 expert nominated genes, the determination of which did not involve the mouse data, receive a uniform increase in priority. If there is additional evidence from the mouse data of relevance of the gene to addiction, the priority is increased further depending on the extent of the evidence, which is measured by the number of mouse phenotypes that link to the gene.

Table S2 shows the distribution of phenotypes that map to mouse genes, both for the entire set of mouse genes considered and for the top 424 genes that were used for our primary analysis of SNP microarrays (these were mapped to human genes via NCBI Homologene). More detailed information on this latter set of genes can be found in supporting file S1 which is discussed in more detail below. Complete details on the data and experiments for this mouse systems genetics project are described in Chesler and colleagues (submitted).

In order to determine the coverage of regions inferred to be undergoing recent adaptive selection [12], [13], all SNPs detected by the LD decay (LDD) test in the Perlegen and HapMap datasets were compared to the Illumina 1M and Affymetrix 6.0 SNPs. Uncovering evidence for recent selection is an additional approach to defining functional human genomic variation. The LDD test identifies alleles undergoing selection by searching for an expected increase with distance in the fraction of inferred recombinant chromosomes surrounding a selected variant. This method is insensitive to local recombination rate because it relies on LD differences between the two alleles at a site, while the local rate influences the extent of LD surrounding both alleles. While over 99.9% of the selected regions defined by the LDD test fall within +/−10 kb of a SNP present on these microarrays, there are some important exceptions. For example, the extensive LD surrounding the selected *DRD4* 7R allele [14] is not captured by these arrays, which contain very few SNPs in the region (only 1 in 100 kb). In general, however, the extensive long-range LD exhibited by these recently selected alleles (up to 1 Mb), and the current density of microarray SNPs, indicates that most of these evolutionarily important alleles can be “tagged” by an adjacent SNP surrogate.

The combined set of 910 genes used for our analysis of SNP microarrays is available as a spreadsheet in supporting file S1. The spreadsheet contains detailed annotation, including the logical category used by the NeuroSNP project, such as “Nicotine System” and “Dopamine System” (further documentation of these categories and other columns is contained in the spreadsheet – see the sheet labeled “Column Descriptions”). Other columns include the Entrez Gene ID and gene symbol, the full name of the gene as well as all known symbol aliases and alternative descriptions, build 36.2 physical mapping data and mouse homologs. Some columns contain links to external databases, such as GenoPedia (http://www.hugenavigator.net/HuGENavigator/startPagePedia.do), which contains a list of all human diseases that have been linked to the gene, including links to publications. The spreadsheet also contains links to the Knowledgebase of Addiction Related Genes (KARG, http://karg.cbi.pku.edu.cn) [15], and also GeneNetwork (http://genenetwork.org) for additional information on mouse systems genetics data. We have also created a web site (http://zork.wustl.edu/nida/neurosnp.html) that contains a searchable database of this set of genes, as well as downloadable files for the gene and SNP databases. These resources will allow investigators to both gather new biologically relevant targets for genetic association studies of addiction, and also to discover new information on well-known targets, such as the extent of tagged coverage in various population by commercial SNP microarrays.

Our complete SNP database is available for download from our web site at http://zork.wustl.edu/nida/neurosnp.html, and the top 5,000 SNPs ranked by GIN prioritization score [4] is provided in a spreadsheet as supporting file S2. The entire database includes all SNPs from dbSNP build 128, and is annotated with allele frequency data from the four HapMap samples; there is no restriction on the allele frequency in the database. There are also flags indicating whether a SNP is on a particular custom microarray specifically designed by Hodgkinson and colleagues to target alcoholism and other addiction related phenotypes [16], or was part of an addiction study by Nielsen and colleagues [17].

## Discussion

We have found that in order to achieve comprehensive tagged coverage of genes that are biologically relevant to addiction in the African, Chinese, European-American and Japanese populations, all the commercial SNP microarrays we considered require significant supplementation. The approaches used here will aid other investigators to supplement these arrays, to target specific genomic regions such as genes and linkage regions, and also improve the general selection of SNPs for genetic studies of addiction based on the “biological role in addiction” criterion. The development of a database of addiction related genes is similar to existing methods and resources in the literature [15]–[17], the primary difference being our development of a SNP database and a prioritization algorithm that allows the systematic supplementation of commercial SNP microarrays.

These methods and resources were developed with the intention that they would be useful to researchers who wish to test *a priori* biological hypotheses, either within the context of a GWAS, or for a more targeted study such as studying specific addiction-related genes or fine mapping a region of genetic linkage for an addiction-related phenotype. The need for this kind of approach has been discussed in the literature [3], and to this end we have used multiple domains to develop a collection of genes with evidence of biological relevance to addiction. The biological principles guiding the selection criteria, such as biochemical pathways and expression data, do not necessarily imply the existence of genetic variants within these genes that influence addiction phenotypes. For example, metabolic pathways related to nicotine are an obvious source for cataloging genes that are biologically relevant to genetic studies of nicotine dependence, but these normal biological systems do not necessarily involve genes with variants that influence abnormal phenotypes. Therefore, the utility of these methods and resources depend on the subjective preferences of investigators on the genetics of addiction and their specific *a priori* biological hypotheses [3].

While the primary utility of the resource we have developed is the supplementation of commercial SNP microarrays, it has several other useful applications. For example, the GIN prioritization scoring method is useful for interpreting the results of a GWAS, and can be used to prioritize SNPs for further study after a GWAS [4], as well as prioritize tests of gene-gene interaction. As the genomic coverage of commercial SNP microarrays improves, and subsequently whole-genome sequencing becomes the new standard, the problem of multiple testing will continue to hinder progress in understanding complex interactions underlying the genetics of addiction and other complex diseases. Therefore tests of gene-gene interaction will in most cases require a mechanism of prioritization, and our database will be a useful resource for this approach. By limiting tests of gene-gene interaction to genes that have a biological connection to the phenotype, the issues of multiple testing and computational tractability are substantially reduced. And even within a set of biologically relevant genes, using the GIN prioritization scores to further refine interactions tests, such as testing only within the top 100 SNPs ranked by these scores, will further reduce the problem.

Table 1 shows that a substantial amount of variation in high priority regions for addiction is currently unaccounted for by these commercial microarrays. Ultimately, the actual number of SNPs used to supplement a microarray will depend on the genotyping platform being used for supplementation. This platform could involve a different technology than that of the original microarray being supplemented. For example, the Affymetrix 6.0 array could be supplemented with custom genotyping on the Illumina GoldenGate platform (http://www.illumina.com), and because some of the SNPs used for Table 1 may not perform well on the GoldenGate platform, the numbers reported in the table may need to be reduced.

Our results highlight the need to supplement commercial SNP microarrays for genetic studies of addiction in order to have adequate coverage for genes in relevant neurobiological pathways. Our SNP database will help researchers to fill the missing gaps by providing a quantitative measure of biological relevance to help prioritize SNPs for supplementation. Addiction researchers should find this resource to be a valuable tool, both in the design and interpretation stages of a GWAS. It helps prioritize coverage of biologically relevant regions, and highlights association signals in those regions when selecting SNPs for replication. It also helps prioritize tests of gene-gene interaction, which can limit multiple testing issues. In this study the focus was on addiction, but our method can be extended to other diseases by creating a new database of biologically relevant genes.

## Methods

We assembled a list of 486 genes biologically relevant for addiction mainly through an expert nomination process (http://grants.nih.gov/grants/guide/notice-files/NOT-DA-07-010.html). Most of these were identified based on involvement in neurobiological pathways relevant to substance abuse and were in part vetted by addiction neurobiologists (see Acknowledgments). These included genes involved in biosynthesis, metabolism, transport, receptor binding, and intracellular signaling. Common biochemical pathways and systems included the serotonergic, noradrenergic, dopaminergic, GABA-ergic, glutamatergic, opioid, alcohol metabolizing and nicotinic systems. In addition, genes involved in the metabolism of FDA-approved medications for substance abuse were also included (e.g., cytochrome p450 genes); genes involved in the medications' pharmacodynamic effects were selected as part of the pathway-based approach. 34 of these genes were added to the initial results of the nomination process because they used for recent custom panel of SNPs for alcoholism and other addiction related traits [16], and 96 of these genes were added from a recent study of addiction [17].

An additional 424 genes were nominated based on behavioral genetic analysis of 255 measures of addiction related phenotypes obtained by the Tennessee Mouse Genome Consortium in the recently expanded panel of over 60 BXD recombinant inbred mouse lines (Chesler and colleagues, submitted). Because we are interested in identifying addiction associated pathway members that are polymorphic in humans, but not necessarily polymorphic in mice, our approach identified both candidate genes for mouse phenotypic variation, and biomolecular correlates of the mouse phenotypes. Each gene was chosen because it either resided in a significant or suggestive QTL interval or was a gene expression correlate of multiple addiction-related phenotypes. The criteria used to place genes on the list included correlations with *p*<.0001, and QTLs, with genome-wide permutation *p*<.05 or *p*<.33, the conventional thresholds for significant and suggestive loci. Convergence of evidence of these effects in multiple addiction assays was the criteria used for list membership. Mouse genes were then mapped to human genes via the HomoloGene database (http://www.ncbi.nlm.nih.gov/sites/entrez?db=homologene).

To explore genetic variation in this combined set of 910 human genes, we created a general purpose genome-wide SNP relational database. The foundation for this database was Build 128 of dbSNP (http://www.ncbi.nlm.nih.gov/projects/SNP), which was our source of physical mapping data and SNP/gene functional properties. We then examined the SNP coverage of these genes as provided by seven commercial microarrays: the Illumina HumanHap300 Duo, HumanHap550, HumanHap650Y and Human1M (http://www.illumina.com), and the Affymetrix Genome-Wide Human SNP Array 5.0 and 6.0 (http://www.affymetrix.com). To assess genomic coverage of common SNPs by these microarrays, we used genotype data for four populations from the International HapMap Project, Public Release 23a (http://www.hapmap.org): African (Yoruba people of Ibidan, Nigeria – YRI), Chinese (Beijing – CHB), European-Americans (CEPH – CEU), and Japanese (Japan – JPT). To estimate LD, we used the program HaploView (version 4.0, http://www.broad.mit.edu/mpg/haploview) [18] to estimate *r* ^2^ for all SNPs within 500 kb of each other. The commonly used condition *r* ^2^≥0.8 was used to assess whether a SNP is tagged through LD in a given population by a given SNP microarray. General database management was done with a combination of SAS [19] and Perl [20].

To provide a mechanism for prioritizing SNPs when supplementing SNP microarrays for addiction, we used the genomic information network (GIN) technique introduced by Saccone and colleagues [4]. The GIN method assigns each SNP a numeric prioritization score indicating the biological relevance for addiction: the higher the score, the greater the priority. Figure S3 shows the network model we used for addiction, which is a modification of the nicotine dependence model used by Saccone and colleagues. The score incorporates a number of factors, including SNP/gene functional properties (such as coding and promoter regions), and evolutionary conserved regions (ECRs, provided by ECRbase [21], http://ecrbase.dcode.org). The original GIN method introduced by Saccone and colleagues incorporated LD into the prioritization score through the use of LD proxies. This is more appropriate when prioritizing SNPs for replication after an initial GWAS. In our current implementation, where we are selecting SNPs to supplement arrays for the discovery phase of a GWAS, we have eliminated the LD component in order to avoid redundancy among the selected SNPs.

The scoring method is identical to Saccone and colleagues [4] for the gene and ECR nodes. The “Addiction Systems” node adds 1 to the score for any of the 486 genes from our expert nomination process. The score for the “Mouse QTL Mapping” node is min(*N*/6,1), where *N* is the number of phenotypes identified for a gene using the systems genetic methods. This means that the score is 1 for all genes where *N* is greater than 6, which corresponds to the top 5% of QTL mapping results. The score is scaled down linearly when *N* is less than 6. Note that this particular GIN model combines information from the expert nomination process and the mouse systems genetics data in the sense that SNPs in the 486 expert nominated genes, the determination of which did not involve the mouse data, would receive an increased score if there was additional evidence from the mouse data of relevance to addiction.

## Supporting Information

Table S1 The coverage of genomic regions biologically relevant to addiction in four commercial SNP microarrays. The table is divided into direct coverage, the percentage of common SNPs actually on the array, and tagged coverage, the percentage of common SNPs tagged by an array through LD at r^2^≥0.8 in the specified HapMap population (for simplicity, we used only two populations). We explore how coverage varies with biological relevance by considering SNPs with a GIN prioritization score greater than a given threshold: the larger the score, the greater the biological relevance. For direct coverage, common SNPs must have a MAF of at least 5% in one of the HapMap populations. For tagged coverage, SNPs must satisfy this condition in the specified population.
(0.06 MB DOC)

Table S2 Results of the systems genetics study to identify mouse genes related to addiction. For each trait the table shows the overall number of genes identified by QTL and gene expression analysis. For our analysis of SNP microarray coverage we used the top 5% from the mouse systems genetics project ranked by the number of phenotypes linked to each gene. The third column shows the number of genes from the top 5% identified for each trait.
(0.06 MB DOC)

Figure S1 The number of genes biologically relevant to addiction that require varying amounts supplementary coverage for the Illumina 610 Quad microarray. Here we consider our primary set of 910 genes. The horizontal axis shows the percentage of SNPs in the gene not tagged by the array in the corresponding population. For example, in the African population, there are 35 genes (3.97%) where at least 90% of the SNPs in those genes are not tagged by this array with r^2^≥0.8 (the rightmost bar in the histogram).
(0.12 MB DOC)

Figure S2 The distribution of the prioritization scores S from the genomic information network (GIN) for addiction. We considered all known SNPs using dbSNP build 128. The score is a cumulative measure of biological relevance based on several factors: our expert nomination process for genes related to addiction, SNP/gene functional properties, human/mouse evolutionary conservation, and mouse QTL mapping methods. For example, SNPs with a score of 0 are not in genes, and are not in LD with a gene or human/mouse evolutionary conserved region with 500 Kb. SNPs in genes have a score of at least 1. The score increases if the gene is biologically relevant to addiction, and increases further depending on the number of mouse QTLs for that gene, and also the functional properties of SNP, such being nonsynonymous or being in a promoter region.
(0.05 MB DOC)

Figure S3 The genomic information network (GIN) model for addiction. The network represents the process of determining a numeric prioritization score for a SNP. The scores are a cumulative measure of biological relevance using SNP/gene functional properties (the Gene node), evolutionary conserved regions (the ECR node), genes biologically relevant to addiction (the Addiction Systems node), and mouse QTL mapping results. The overall GIN prioritization scores can be used to prioritize SNPs when supplementing commercial microarrays for addiction.
(0.05 MB DOC)

File S1 This workbook contains two sheets: (1) an annotated sheet with the primary set of 910 genes used for analysis (2) a list of column descriptions
(0.95 MB XLS)

File S2 This workbook contains two sheets: (1) an annotated sheet with the top 5,000 SNPs ranked by prioritization score (2) a list of column descriptions
(3.76 MB XLS)
