# Supplementary material for: Supplementing High-Density SNP Microarrays for Additional Coverage of Disease-Related Genes: Addiction as a Paradigm
Source: PLoS One. 2009 Apr 21;4(4):e5225. doi: 10.1371/journal.pone.0005225 (PMC2668711; doi:10.1371/journal.pone.0005225)
Supplement: Table S1 — The coverage of genomic regions biologically relevant to addiction in four commercial SNP microarrays. The table is divided into direct coverage, the percentage of common SNPs actually on the array, and tagged coverage, the percentage of common SNPs tagged by an array through LD at r2≥0.8 in the specified HapMap population (for simplicity, we used only two populations). We explore how coverage varies with biological relevance by considering SNPs with a GIN prioritization score greater than a given threshold: the larger the score, the greater the biological relevance. For direct coverage, common SNPs must have a MAF of at least 5% in one of the HapMap populations. For tagged coverage, SNPs must satisfy this condition in the specified population. (0.06 MB DOC) [file pone.0005225.s003.doc]

**Table S1.** The coverage of genomic regions biologically relevant to addiction in four commercial SNP microarrays. The table is divided into direct coverage, the percentage of common SNPs actually on the array, and tagged coverage, the percentage of common SNPs tagged by an array through LD at *r*^2^ ≥ 0.8 in the specified HapMap population (for simplicity, we used only two populations). We explore how coverage varies with biological relevance by considering SNPs with a GIN prioritization score greater than a given threshold: the larger the score, the greater the biological relevance . For direct coverage, common SNPs must have a MAF of at least 5% in one of the HapMap populations. For tagged coverage, SNPs must satisfy this condition in the specified population.

|  |  | *SNP Microarray Coverage (%)* | | | |
| --- | --- | --- | --- | --- | --- |
| Prioritization Score Threshold *T* | Common SNPs with Score ≥ *T* | Affymetrix 5.0 | Affymetrix 6.0 | Illumina 610 Quad | Illumina 1M |
|  |  | *Direct Coverage* | | | |
| 0.0 | 3,134,712 | 14 | 28 | 18 | 29 |
| 0.5 | 1,227,957 | 14 | 29 | 20 | 35 |
| 1.0 | 1,199,104 | 14 | 29 | 20 | 34 |
| 1.5 | 327,970 | 14 | 28 | 21 | 38 |
| 2.0 | 134,439 | 14 | 29 | 23 | 39 |
| 2.5 | 23,382 | 13 | 28 | 26 | 43 |
| 3.0 | 2,018 | 11 | 27 | 32 | 57 |
| 3.5 | 133 | 14 | 30 | 43 | 78 |
|  |  | *Tagged Coverage – African* | | | |
| 0.0 | 2,498,196 | 44 | 69 | 58 | 73 |
| 0.5 | 973,372 | 45 | 69 | 60 | 76 |
| 1.0 | 950,613 | 45 | 69 | 60 | 75 |
| 1.5 | 258,641 | 44 | 68 | 59 | 76 |
| 2.0 | 105,752 | 43 | 68 | 59 | 75 |
| 2.5 | 18,309 | 40 | 64 | 58 | 73 |
| 3.0 | 1,543 | 41 | 66 | 60 | 82 |
| 3.5 | 96 | 35 | 60 | 62 | 90 |
|  |  | *Tagged Coverage – European-American* | | | |
| 0.0 | 2,272,199 | 66 | 85 | 89 | 93 |
| 0.5 | 883,919 | 66 | 85 | 91 | 94 |
| 1.0 | 862,792 | 66 | 85 | 91 | 94 |
| 1.5 | 234,590 | 66 | 85 | 91 | 94 |
| 2.0 | 96,719 | 64 | 84 | 91 | 94 |
| 2.5 | 16,878 | 60 | 81 | 90 | 93 |
| 3.0 | 1,401 | 63 | 83 | 91 | 96 |
| 3.5 | 82 | 59 | 79 | 89 | 98 |
