# Supplementary material for: Supplementing High-Density SNP Microarrays for Additional Coverage of Disease-Related Genes: Addiction as a Paradigm
Source: PLoS One. 2009 Apr 21;4(4):e5225. doi: 10.1371/journal.pone.0005225 (PMC2668711; doi:10.1371/journal.pone.0005225)
Supplement: Table S2 — Results of the systems genetics study to identify mouse genes related to addiction. For each trait the table shows the overall number of genes identified by QTL and gene expression analysis. For our analysis of SNP microarray coverage we used the top 5% from the mouse systems genetics project ranked by the number of phenotypes linked to each gene. The third column shows the number of genes from the top 5% identified for each trait. (0.06 MB DOC) [file pone.0005225.s004.doc]

**Table S2.** Results of the systems genetics study to identify mouse genes related to addiction. For each trait the table shows the overall number of genes identified by QTL and gene expression analysis. For our analysis of SNP microarray coverage we used the top 5% from the mouse systems genetics project ranked by the number of phenotypes linked to each gene. The third column shows the number of genes from the top 5% identified for each trait.

| Trait | Genes | Top Genes Only |
| --- | --- | --- |
| Zero-maze time open | 2,951 | 84 |
| Zero-maze latency | 2,069 | 52 |
| Adrenal Weight | 1,581 | 44 |
| Total activity count, number of beam breaks | 1,326 | 28 |
| Rears (vertical beam breaks) from 0 to 15 min | 1,193 | 30 |
| Locomotion (cM) from 0 to 15 min | 1,142 | 25 |
| Suppression of activity in altered context | 1,044 | 35 |
| Activity in altered context during presentation of cue | 957 | 28 |
| Adult neurogenesis rostral migratory stream | 931 | 21 |
| Locomotion (cM) from 15 to 30 min | 923 | 30 |
| Cocaine-induced conditioned place preference (CPP) | 907 | 24 |
| Total locomotion (cM in 1 hr) in the periphery | 886 | 29 |
| Locomotion (cM) from 0 to 15 min | 874 | 22 |
| Zero-Maze open time | 788 | 31 |
| Locomotion (cM) from 15 to 30 min | 785 | 23 |
| Morphine total rears | 737 | 26 |
| Cocaine locomotor activity | 734 | 24 |
| Rears (vertical beam breaks) from 0 to 15 min in the periphery | 728 | 36 |
| Total distance traveled | 648 | 27 |
| Zero-maze time closed | 646 | 26 |
| Morphine induced salivation | 642 | 9 |
| Total rears (vertical beam breaks) | 613 | 27 |
| Morphine induced defecation | 586 | 18 |
| Zero-maze time closed | 583 | 24 |
| Time below threshold | 581 | 18 |
| Maximum startle response at 80 db | 576 | 21 |
| Zero-maze percentage open | 574 | 25 |
| Prepulse Inhibition at 85 db | 573 | 17 |
| Morphine total locomotion | 571 | 29 |
| Prepulse Inhibition at 80 db | 571 | 19 |
| Maximum startle response at 85 db | 561 | 22 |
| Difference in percentage time spent from preconditioning | 491 | 14 |
| Contextual activity | 475 | 12 |
| Locomotion in the periphery as a function of total locomotion. | 429 | 7 |
| Open field vertical activity count | 371 | 13 |
| Transitions between light and dark compartments | 347 | 14 |
| Percentage of maximum startle response at 85 db | 274 | 7 |
| Percentage of maximum startle response at 80 db | 206 | 6 |
| Latency for tail clip procedure | 147 | 2 |
| Time in seconds spent on the drug paired (white) compartment at baseline, prior to any conditioning | 139 | 3 |
| Total locomotion (cM in 1 hr) | 136 | 2 |
| Average of two trails for the hot plate procedure | 135 | 7 |
| Total locomotion (activity beam breaks) in the center + periphery | 113 | 4 |
| Average of all six trails for left and right hind paws in Hargreave's procedure | 88 | 1 |
| Average of three trails for tail withdrawal procedure | 54 | - |
| Average Vonfrey threshold for both paws for days 1 and 2 | 13 | - |
|  |  |  |
