# Supplementary material for: Supplementing High-Density SNP Microarrays for Additional Coverage of Disease-Related Genes: Addiction as a Paradigm
Source: PLoS One. 2009 Apr 21;4(4):e5225. doi: 10.1371/journal.pone.0005225 (PMC2668711; doi:10.1371/journal.pone.0005225)
Supplement: Figure S1 — The number of genes biologically relevant to addiction that require varying amounts supplementary coverage for the Illumina 610 Quad microarray. Here we consider our primary set of 910 genes. The horizontal axis shows the percentage of SNPs in the gene not tagged by the array in the corresponding population. For example, in the African population, there are 35 genes (3.97%) where at least 90% of the SNPs in those genes are not tagged by this array with r2≥0.8 (the rightmost bar in the histogram). (0.12 MB DOC) [file pone.0005225.s005.doc]

| 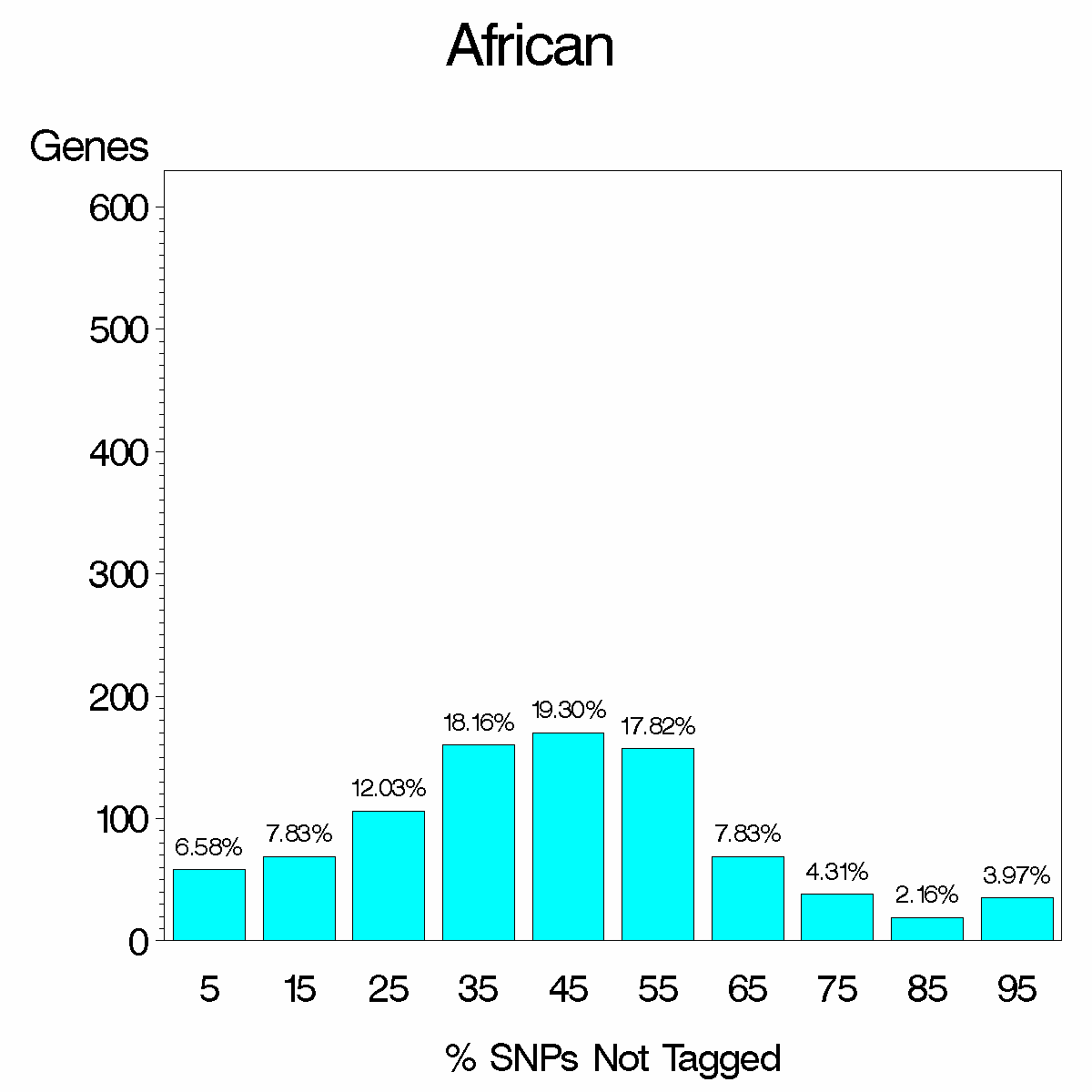 | 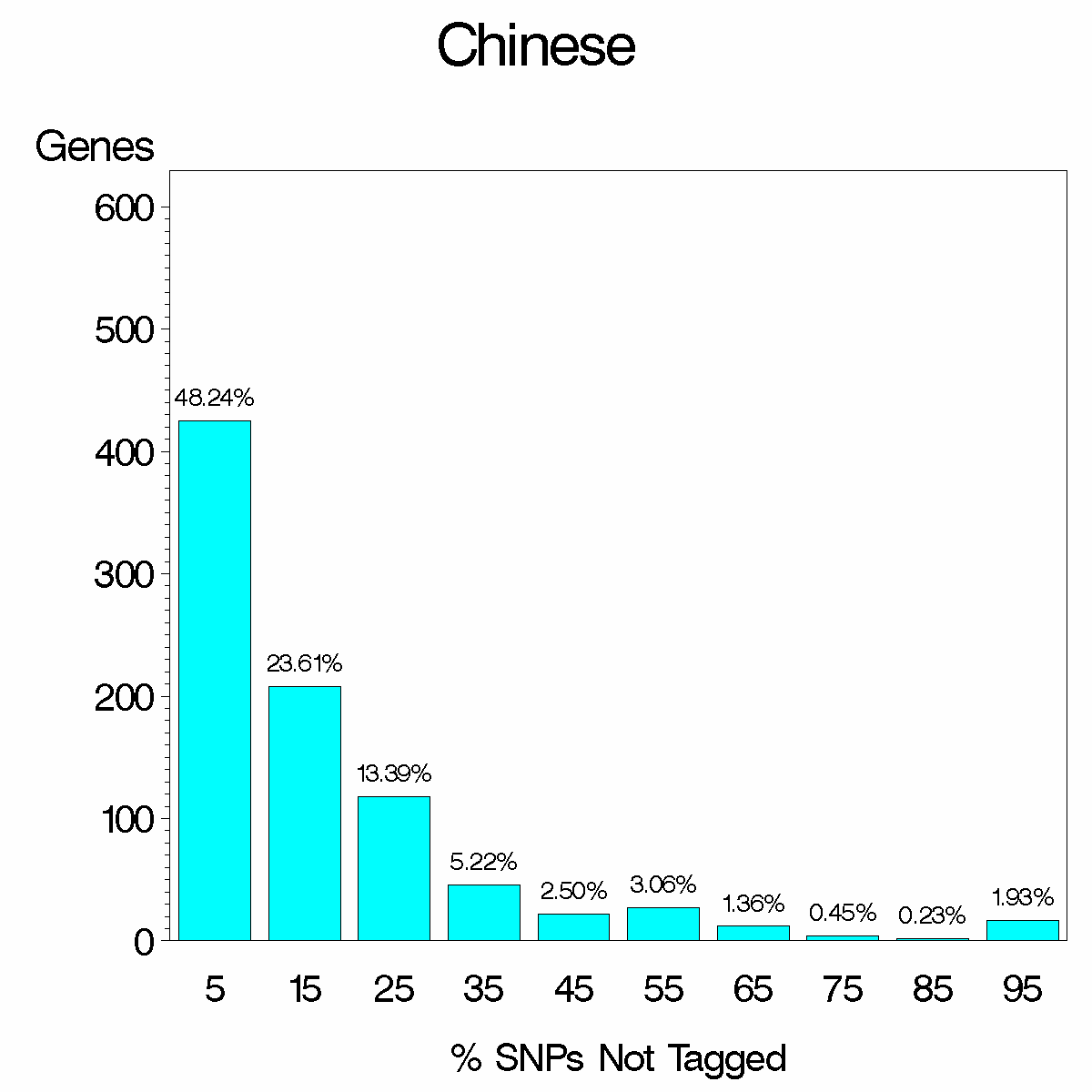 |
| --- | --- |
| 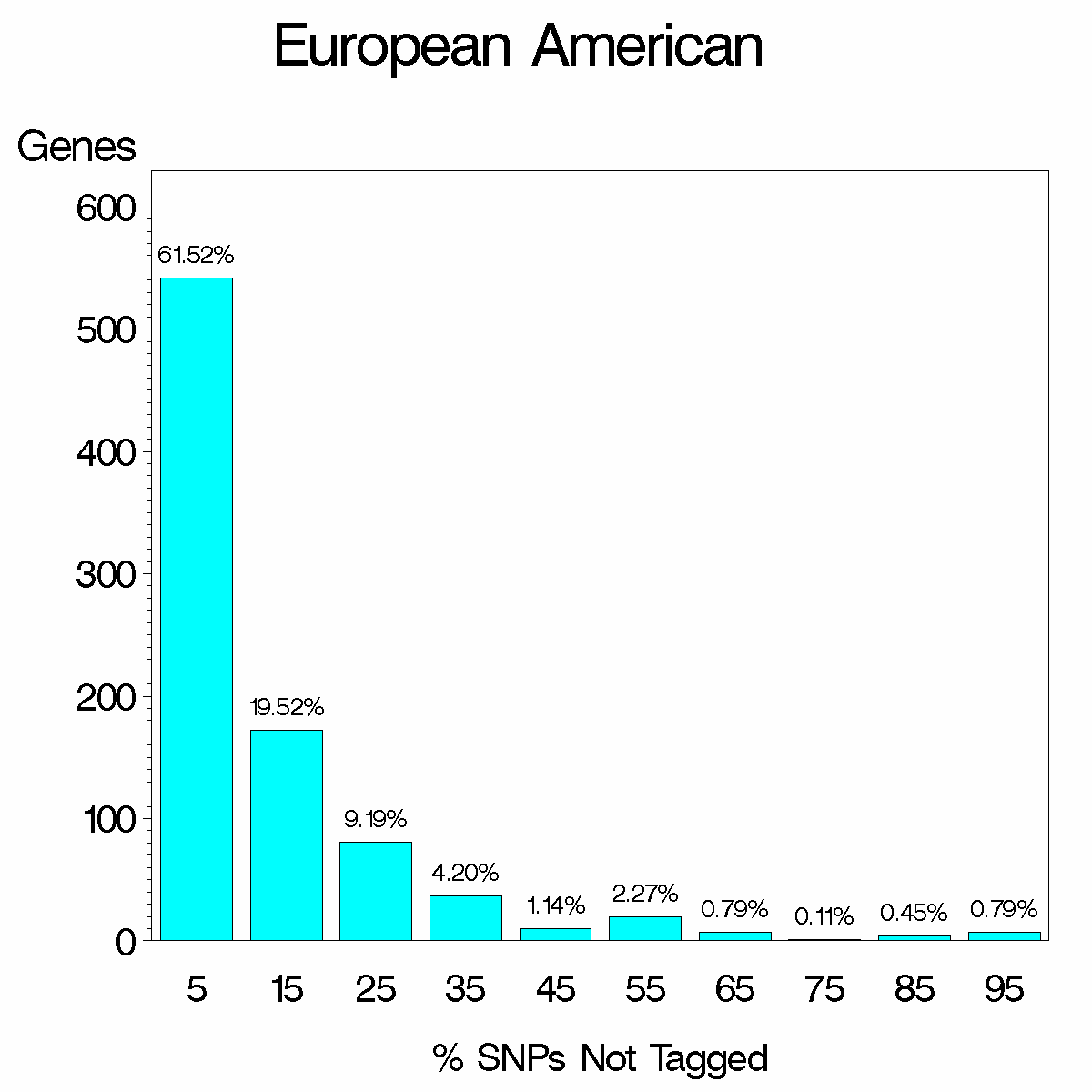 | 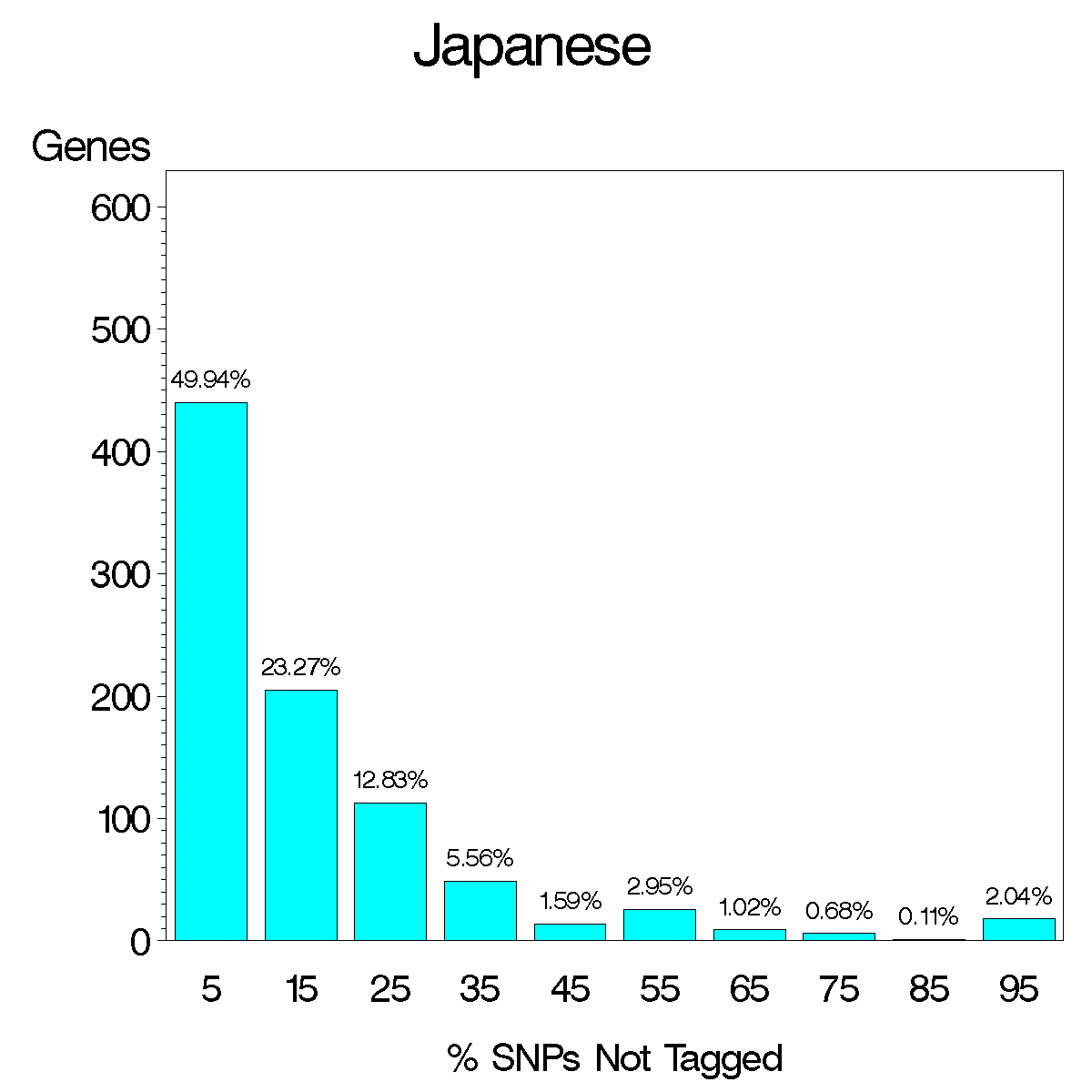 |

**Figure S1.** The number of genes biologically relevant to addiction that require varying amounts supplementary coverage for the Illumina 610 Quad microarray. Here we consider our primary set of 910 genes. The horizontal axis shows the percentage of SNPs in the gene not tagged by the array in the corresponding population. For example, in the African population, there are 35 genes (3.97%) where at least 90% of the SNPs in those genes are not tagged by this array with *r*^2^ ≥ 0.8 (the rightmost bar in the histogram).
