# Supplementary material for: Supplementing High-Density SNP Microarrays for Additional Coverage of Disease-Related Genes: Addiction as a Paradigm
Source: PLoS One. 2009 Apr 21;4(4):e5225. doi: 10.1371/journal.pone.0005225 (PMC2668711; doi:10.1371/journal.pone.0005225)
Supplement: Figure S2 — The distribution of the prioritization scores S from the genomic information network (GIN) for addiction. We considered all known SNPs using dbSNP build 128. The score is a cumulative measure of biological relevance based on several factors: our expert nomination process for genes related to addiction, SNP/gene functional properties, human/mouse evolutionary conservation, and mouse QTL mapping methods. For example, SNPs with a score of 0 are not in genes, and are not in LD with a gene or human/mouse evolutionary conserved region with 500 Kb. SNPs in genes have a score of at least 1. The score increases if the gene is biologically relevant to addiction, and increases further depending on the number of mouse QTLs for that gene, and also the functional properties of SNP, such being nonsynonymous or being in a promoter region. (0.05 MB DOC) [file pone.0005225.s006.doc]

**
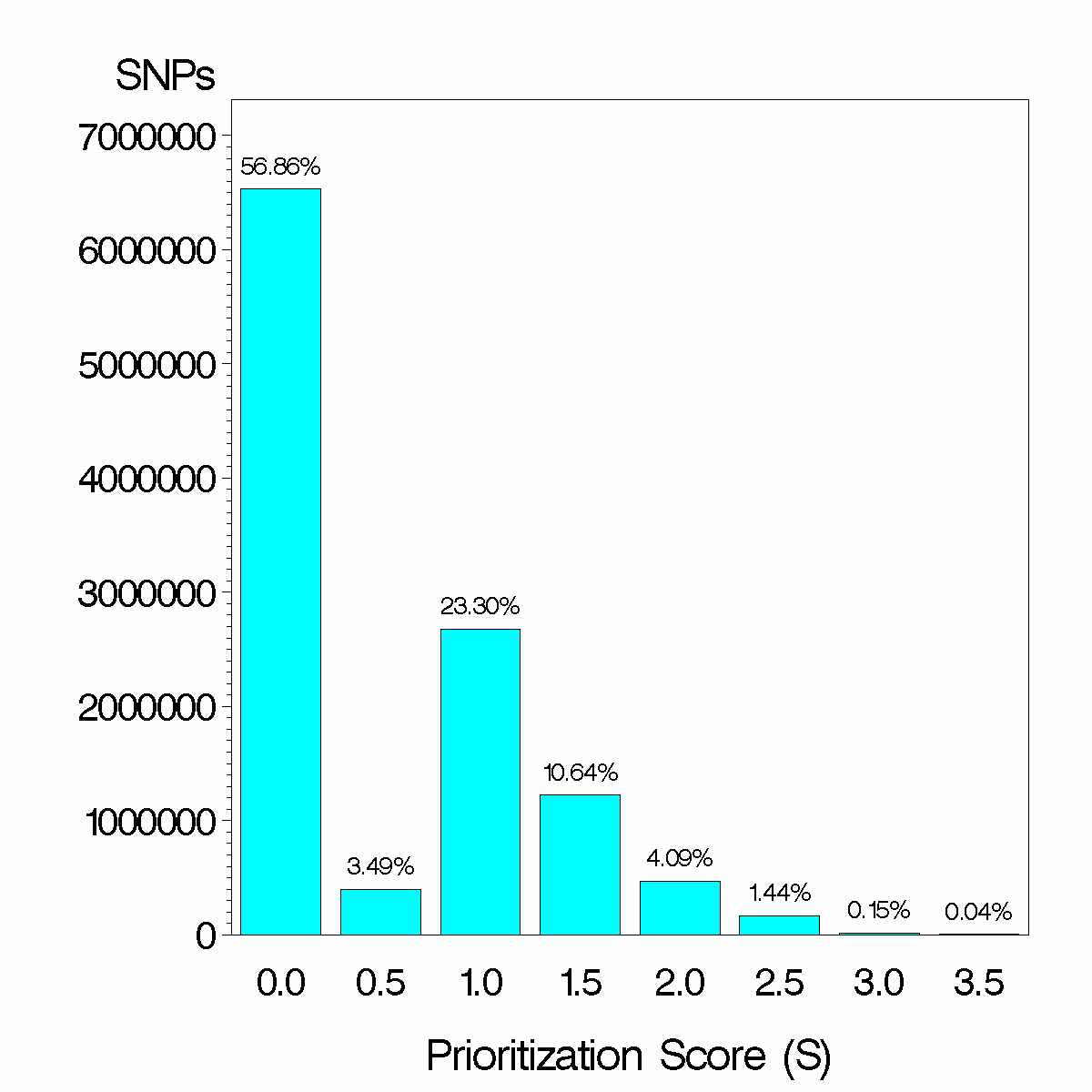
**

**Figure S2.** The distribution of the prioritization scores *S* from the genomic information network (GIN) for addiction. We considered all known SNPs using dbSNP build 128. The score is a cumulative measure of biological relevance based on several factors: our expert nomination process for genes related to addiction, SNP/gene functional properties, human/mouse evolutionary conservation, and mouse QTL mapping methods. For example, SNPs with a score of 0 are not in genes, and are not in LD with a gene or human/mouse evolutionary conserved region with 500 Kb. SNPs in genes have a score of at least 1. The score increases if the gene is biologically relevant to addiction, and increases further depending on the number of mouse QTLs for that gene, and also the functional properties of SNP, such being nonsynonymous or being in a promoter region.
