# Supplementary material for: Supplementing High-Density SNP Microarrays for Additional Coverage of Disease-Related Genes: Addiction as a Paradigm
Source: PLoS One. 2009 Apr 21;4(4):e5225. doi: 10.1371/journal.pone.0005225 (PMC2668711; doi:10.1371/journal.pone.0005225)
Supplement: Figure S3 — The genomic information network (GIN) model for addiction. The network represents the process of determining a numeric prioritization score for a SNP. The scores are a cumulative measure of biological relevance using SNP/gene functional properties (the Gene node), evolutionary conserved regions (the ECR node), genes biologically relevant to addiction (the Addiction Systems node), and mouse QTL mapping results. The overall GIN prioritization scores can be used to prioritize SNPs when supplementing commercial microarrays for addiction. (0.05 MB DOC) [file pone.0005225.s007.doc]

**
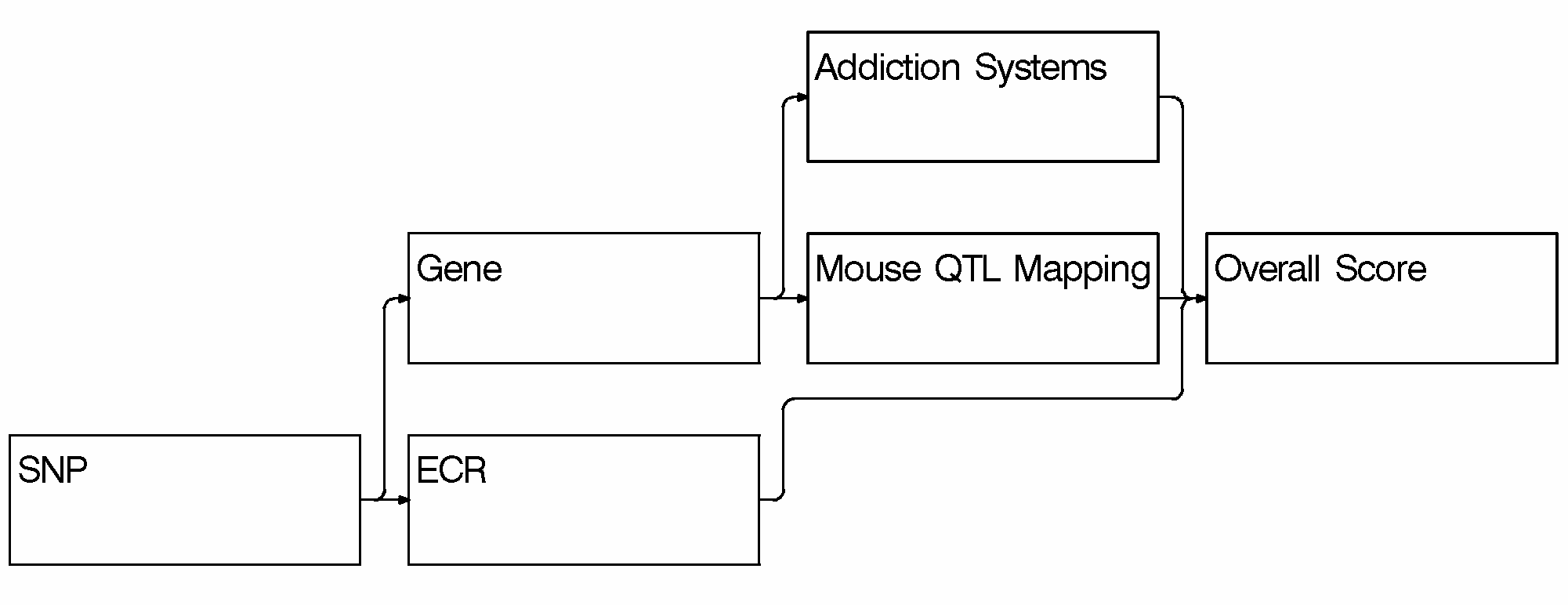
**

**Figure S3.** The genomic information network (GIN) model for addiction. The network represents the process of determining a numeric prioritization score for a SNP. The scores are a cumulative measure of biological relevance using SNP/gene functional properties (the Gene node), evolutionary conserved regions (the ECR node), genes biologically relevant to addiction (the Addiction Systems node), and mouse QTL mapping results. The overall GIN prioritization scores can be used to prioritize SNPs when supplementing commercial microarrays for addiction.
